# Challenges of using asthma admission rates as a measure of primary care quality in children: An international comparison

**DOI:** 10.1177/13558196211012732

**Published:** 2021-07-28

**Authors:** Irina Lut, Kate Lewis, Linda Wijlaars, Ruth Gilbert, Tiffany Fitzpatrick, Hong Lu, Astrid Guttmann, Sharon Goldfield, Shaoke Lei, Geir Gunnlaugsson, Stefán Hrafn Jónsson, Reli Mechtler, Mika Gissler, Anders Hjern, Pia Hardelid

**Affiliations:** 1 PhD Student, UCL Great Ormond Street Institute of Child Health, UK; 2 PhD Student, UCL Great Ormond Street Institute of Child Health, UK; 3 Senior Research Associate, UCL Great Ormond Street Institute of Child Health, UK; 4 Professor, UCL Great Ormond Street Institute of Child Health, UK; 5 Epidemiologist, Child Health Evaluative Sciences, Hospital for Sick Children, Canada; 6 Data Analyst, ICES, Canada; 7 Professor, ICES & Dalla Lana School of Public Health, University of Toronto, Canada; 8 Professor, Murdoch Children’s Research Institute & Division of Medicine, Dentistry and Health Sciences, University of Melbourne, Australia; 9 Data Analyst, Murdoch Children’s Research Institute, Australia; 10 Professor, School of Social Sciences, University of Iceland, Iceland; 11 Professor, School of Social Sciences, University of Iceland, Iceland; 12 Doctor, Johannes Kepler University, Austria; 13 Professor, Finnish Institute for Health and Welfare, Finland and Department of Neurobiology, Care Sciences and Society, Karolinska Institute, Sweden; 14 Professor, Department of Public Health Sciences, Stockholm University, Sweden; 15 Department of Clinical Epidemiology, Karolinska Institutet, Sweden; 16 Associate Professor, UCL Great Ormond Street Institute of Child Health, UK

**Keywords:** Asthma, primary care, paediatrics

## Abstract

**Objectives:**

To demonstrate the challenges of interpreting cross-country comparisons of paediatric asthma hospital admission rates as an indicator of primary care quality.

**Methods:**

We used hospital administrative data from >10 million children aged 6–15 years, resident in Austria, England, Finland, Iceland, Ontario (Canada), Sweden or Victoria (Australia) between 2008 and 2015. Asthma hospital admission and emergency department (ED) attendance rates were compared between countries using Poisson regression models, adjusted for age and sex.

**Results:**

Hospital admission rates for asthma per 1000 child-years varied eight-fold across jurisdictions. Admission rates were 3.5 times higher when admissions with asthma recorded as any diagnosis were considered, compared with admissions with asthma as the primary diagnosis. Iceland had the lowest asthma admission rates; however, when ED attendance rates were considered, Sweden had the lowest rate of asthma hospital contacts.

**Conclusions:**

The large variations in childhood hospital admission rates for asthma based on the whole child population reflect differing definitions, admission thresholds and underlying disease prevalence rather than primary care quality. Asthma hospital admissions among children diagnosed with asthma is a more meaningful indicator for inter-country comparisons of primary care quality.

## Introduction

Inter-country comparisons of health indicators are a powerful tool in health research. Key health indicators such as infant mortality and life expectancy at birth are used by international government organisations and research funders to monitor the state of nations’ health and compare health systems between countries.^1^

Since countries with similar levels of national income and comparable health systems could be expected to have analogous health outcomes, any observed differences between such countries could be due to the organisation or delivery of health care. The Organisation of Economic Co-operation and Development (OECD)^2^ and the World Health Organisation European Region^3^ have published international comparisons of hospital admission rates for ambulatory care sensitive conditions (ACSC). For chronic ACSC, including asthma and diabetes, exacerbations requiring hospital admission are thought to be preventable through proactive management in primary care.^4^ International comparisons of hospital admission rates for ACSCs may therefore reflect differences in access to, or quality of, primary care services between countries.

Asthma is the most common chronic condition among children,^5^ with an estimated global prevalence of 13% in children aged 13–14 years.^6^ Asthma is primarily managed in primary care using pharmacological interventions.^7^ It is well established that pharmacological treatment, in combination with other primary care interventions, including good inhaler technique, self-management education, regular reviews and personalized action plans, can reduce severe asthma complications and related hospital admissions. Asthma admissions among children and adults are therefore monitored by health care observatories and providers^8,9^ as an indicator of primary care quality. Consequently, there is also interest in using international comparisons of asthma admission rates to compare the quality of primary care for children with chronic conditions between countries.^10,11^

A number of factors may affect asthma admission rates. The prevalence and severity of asthma in children vary across countries. Indoor and outdoor environments, including exposure to environmental tobacco smoke, quality of housing, and ambient air pollution levels vary both within and between jurisdictions and may lead to differences in the rate of acute asthma exacerbations.^12–14^ Further, hospital bed occupancy, the use of acute/medical assessment units, clinical coding practices and admission thresholds are key determinants of hospital admission rates, which may also be influenced by local or national secondary care policies, such as maximum waiting times in emergency departments.^15,16^

Our aim is to highlight challenges in interpreting inter-country comparisons of paediatric asthma hospital admission rates as indicators of the quality of primary care for children with chronic conditions. We focus on how comparisons based on asthma admission rates per population, calculated using administrative hospital data from multiple jurisdictions, may be interpreted differently depending on the definition of ‘asthma admission’ used. Further, we consider how the observed association between asthma prevalence and admission rates affect our interpretation of cross-country comparisons of asthma hospitalisation rates, and in turn, asthma admission rates as an indicator of primary care quality.

## Methods

### Data sources and study population

Seven jurisdictions (five countries and two provinces/states) contributed administrative hospital data on asthma admissions: Austria, England, Finland, Iceland, Province of Ontario (Canada), Sweden, and State of Victoria (Australia) – see Online Supplement, Table S1. These jurisdictions all have universal health care services for children, but different health care financing and primary care delivery models (Table 1). The hospital admission data included all hospital episodes that met the definition of an admission in a particular jurisdiction. All jurisdictions used the International Classification of Diseases, version 10 (ICD-10), for coding in their respective administrative hospital databases. Data for the last five years available were requested from each jurisdiction. This produced data for the period 2008 and 2015, although 2012 was the most recent year for which data were available for all jurisdictions (Table 2).

**Table 1.** Summary of health system, primary care organization model, and asthma care pathway for children by jurisdiction.^17^

Jurisdiction	Type of health care system^18^	Model of primary care organization^19^	Asthma care pathway for children (summary)
Austria	Social health insurance	Professional non-hierarchical^a^	• Children with asthma are primarily diagnosed and managed in primary care by GPs and/or paediatrician. • Children with severe asthma are usually managed by a pulmonologist as an outpatient.
England	National health service	Professional hierarchical gatekeeper^b^	• Children and adults with asthma are diagnosed and managed in primary care.^20^ • Children with difficult to control asthma should be referred by the treating GP to a specialist GP, nurse or respiratory consultant.^4^ • Some areas of England operate specialist asthma centres with multidisciplinary teams for children with severe asthma^21^
Finland	National health service	Public hierarchical normative^c^	• Children aged 0-6 years with asthma are diagnosed and treated in specialized health care.^22^ • Children aged 7-17 years and adults with asthma are diagnosed and treated in primary care.^22^ • Asthma qualifies for special reimbursement for medication, given by the National Social Insurance Institution.^23^
Iceland	National health service	Public hierarchical normative	• Primary health care services with GPs, delivered in state-run health centres in across the country, with access 24/7, depending on location. Free of charge to children less than 18 years. • Private specialist consultations are free of charge with a referral note from a GP, and for children less than 2 years old; without a referral note from a GP, subsidized service-for-fee (40 to 50 euros), with threshold for maximum cost of about 350 euros per family per year. • Hospital services (larger cities) are free-of-charge
Ontario (Canada)	National health service (provincial jurisdiction)	Mix of free professional non-hierarchical and professional hierarchical gatekeeper	• Children with asthma are primarily diagnosed and managed in primary care by GPs and/or paediatricians although specialized asthma clinics are available for those with frequent hospital use or severe disease. • No standardized pathway of outpatient asthma care; hospital guidelines for referrals to specialized care have been developed provincially. • Medication (e.g. inhalers) are publicly funded for children whose families are on social assistance and those with high prescription costs relative to family income.
Sweden	National health service	Public hierarchical normative	• Children with asthma are primarily diagnosed and managed in primary care, but children with severe asthma are usually managed in paediatric specialist outpatient care.
Victoria (Australia)	National Health Service	Professional hierarchical gatekeeper	Children are diagnosed and managed (regular monitoring and reviews) in primary care. A referral should be made to a specialist if: • there is an unclear response to asthma treatment/asthma not controlled • before prescribing high-dose inhaled corticosteroids in children aged 5 and under

^a^The professional non-hierarchical model refers to a system in which primary care is provided by health care professionals without strong regulation from the state or insurance funds. Professionals are self-employed and work in competition to each other.^19^

^b^The professional hierarchical gatekeeper model refers to a system in which independent physicians are themselves accountable for the management of resources used for health care.^19^

^c^The public hierarchical normative model refers to a system in which primary care has a central place in the health care system and is run by the state rather than professionals.^19^

**Table 2.** Number of asthma admissions (with asthma as a primary diagnosis) and rates, denominator populations.

Jurisdiction (years of data on which admission numbers and rates are based)	Number of hospital admissions for asthma 6–15 years	Number of child-years 6–15 years	Crude asthma admission rate/1000 child years	Age/sex standardized rate/1000 child years	ISAAC asthma prevalence 6–7 years^a^ (%)	ISAAC asthma prevalence 13–14 years^a^ (%)
Austria (2010–2014)	2,829	4,202,689	0.67	0.68	4.2	7.0
England (2009–2013)	57,997	30,436,521	1.91	1.91	26.8	25.1
Finland (2009–2013)	1,129	2,961,920	0.38	0.38	–	7.7
Iceland (2011–2015)	18	210,270	0.09	0.09	–	–
Ontario (2009–2015)	5,657	11,022,428	0.51	0.52	19.0	16.3
Sweden (2008–2012)	1,207	4,519,970	0.27	0.26	9.3	12.0
Victoria (2000–2012)	5,845	2,672,553	2.19	2.16	25.5	37.3

^a^ISAAC wave 3 data collection occurred between 2001–2003.

Denominator populations of resident children were obtained from the statistical agencies of each jurisdiction, apart from in Ontario where numbers of resident children were extracted from the Registered Persons Database, a population-based registry of all legal Ontario residents eligible for provincial health insurance (Table S1).

As there is no ongoing, universally collected data source on asthma prevalence that is comparable across the jurisdictions of study, asthma prevalence estimates for each jurisdiction were obtained from International Study of Asthma and Allergy in Children (ISAAC) Wave 3. These data were collected between 2001 and 2003,^6^ and published for two age groups: 6–7 years and 13–14 years, for boys and girls combined.

### Outcomes

We examined asthma admissions among children aged 6 to 15 years old inclusive. From each jurisdiction, we obtained aggregate data on the number of inpatient hospital admissions that had a primary diagnosis of asthma (ICD-10 J45 and J46) in the target age group (see Table S1 for definitions of a hospital admission and ‘primary diagnosis’ used in each jurisdiction). We also obtained admissions where asthma was recorded as any diagnosis (that is, either the primary or one of the secondary diagnoses). To ensure that the admissions were not due to other chronic respiratory conditions, we excluded any asthma admissions where a code indicating cystic fibrosis (ICD-10 E84), other lung disorders (J984), chronic respiratory diseases originating in the perinatal period (P27, P28), congenital anomalies of the respiratory system (Q30, Q31, Q32, Q33, Q34), congenital tracheo-oesophageal fistula (Q391, Q392) or congenital malformations of aorta (Q254) was also recorded, following the definition used by the Agency for Healthcare Research and Quality.^24^ Where available (for Iceland, Sweden and Ontario), we obtained data on emergency department (ED) attendances where asthma was recorded as the primary diagnosis.

### Statistical analyses

Data were split by sex and into three age groups (6–9 years, 10–12 years and 13–15 years).

We calculated hospital admission rates with asthma as a primary diagnosis per 1000 child-years by age group, sex, year of admission and jurisdiction, with 95% confidence intervals. Age-sex standardized admission rates were calculated using direct standardization; we derived a standard population based on the sum of age and sex specific denominator populations across the seven jurisdictions. We used Poisson regression models, adjusted for age group and sex, to calculate incidence rate ratios (IRRs) to compare asthma admission rates, where asthma was recorded as the primary diagnosis, between countries; Sweden was chosen as the baseline country. We repeated these analyses for asthma recorded as any (primary or additional) diagnoses, to examine if the pattern by jurisdiction was robust to differing definitions of an ‘asthma admission’.

We fitted Poisson regression models adjusted for age group and sex to compare ED attendance rates between jurisdictions. Examining ED attendances and admission rates provides an indication of the differences in admission thresholds between jurisdictions.

Hospital admissions in Austria, Finland, Iceland and Sweden were defined as requiring an overnight stay in hospital (Table S1). To explore the impact of using similar definitions of ‘hospital admission’ across countries, we excluded 0-day admissions in England and recalculated admission rates. Note that as actual times of admission and discharge were not available, despite excluding 0-day admissions, we could not rule out that some non-overnight stays were still included. It was not possible to exclude 0-day admissions in Victoria or Ontario.

We established the association between asthma prevalence rates from ISAAC (based on the proportion of children who reported ever having had asthma in their lifetime) and asthma admission rates by fitting a further Poisson regression model. For these analyses we used the age groups 6–7 years and 13–14 years for the year 2010, which was the earliest year of data available in the countries for which ISAAC data were also available. In this model, asthma admission count was the outcome, the ISAAC prevalence of asthma (‘asthma ever’) was the exposure variable, and the population denominator (for the admission rates) was included as the offset. Separate models were fitted for 6–7 and 13–14 year-olds, respectively. We tested whether model fit improved when including a quadratic term for asthma prevalence using likelihood ratio (LR) tests, where an LR test *p*-value < 0.05 was taken as evidence that inclusion of a quadratic term improved model fit. We compared the observed admission rates against the expected rates for each jurisdiction derived from the final model, to graphically assess the association between asthma prevalence and admission rates. All analyses were carried out in Stata version 15 and graphics created in Microsoft Excel.

## Results

**Figure 1.**
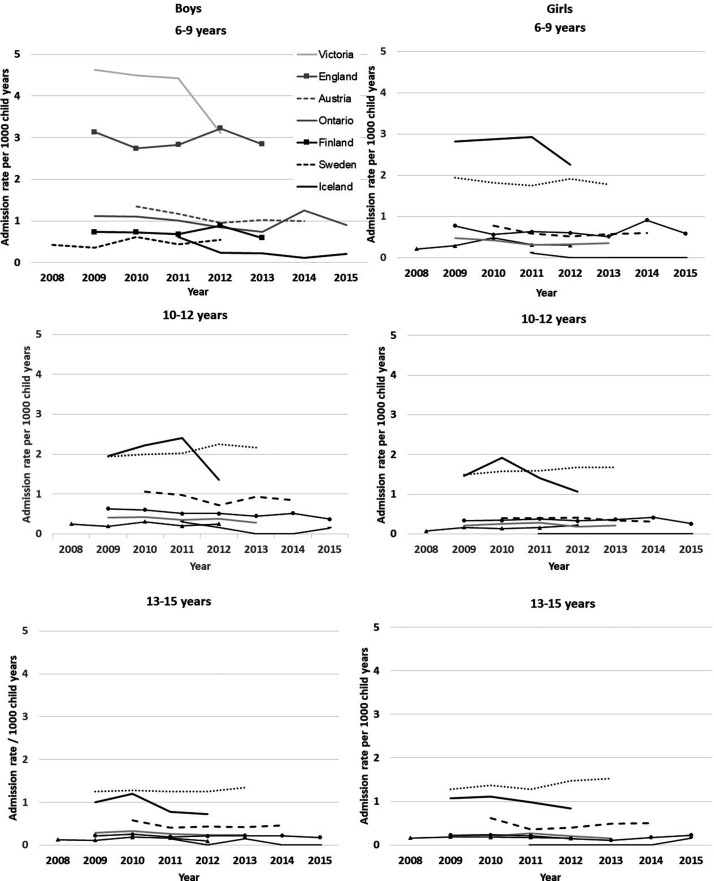
Admission rates with asthma as the primary diagnosis (per 1000 child years) by age, sex, jurisdiction and year.

This study included 74,682 asthma admissions during 56,026,351 child-years (Table 2). In 2012, the study population included 10,701,913 resident children. Overall, asthma hospital admission rates varied eight-fold between jurisdictions (Figure 1), with Iceland having the lowest age-sex standardized rate, 0.09 per 1000 child-years, and Victoria the highest, 2.19 per 1000 child-years (Table 2). After Iceland, Sweden had the second lowest admission rate, 0.27 per 1000 child-years (Table 2).

**Figure 2.**
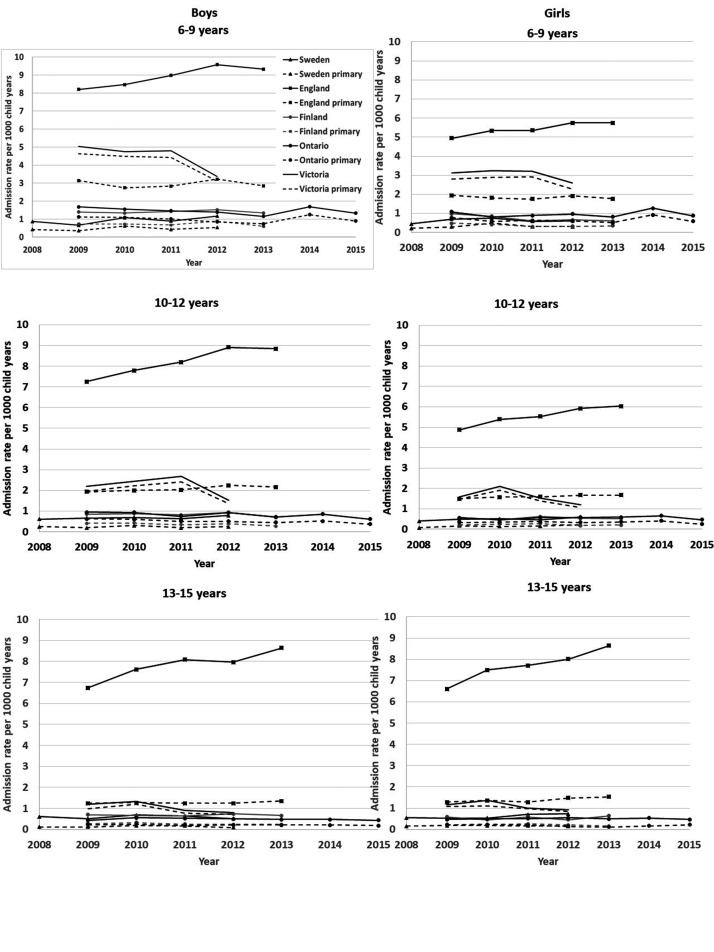
Asthma, as any vs primary diagnosis, admission rates by age, sex, jurisdiction and year.

Admission rates were 3.38 times higher when admissions with any asthma diagnosis was used to calculate admission rates (95% CI: 3.35–3.41; Figure 2), compared with asthma as primary diagnosis only. The differences were largest for 13–15 year-olds. England’s admission rate with asthma as a primary diagnosis was seven times higher than Sweden’s (IRR = 7.19; 95% CI: 6.79–7.61), but eleven times higher when using asthma recorded as any diagnosis (IRR = 10.84; 95% CI: 10.45–11.23; Figure 2, Table S2).

**Figure 3.**
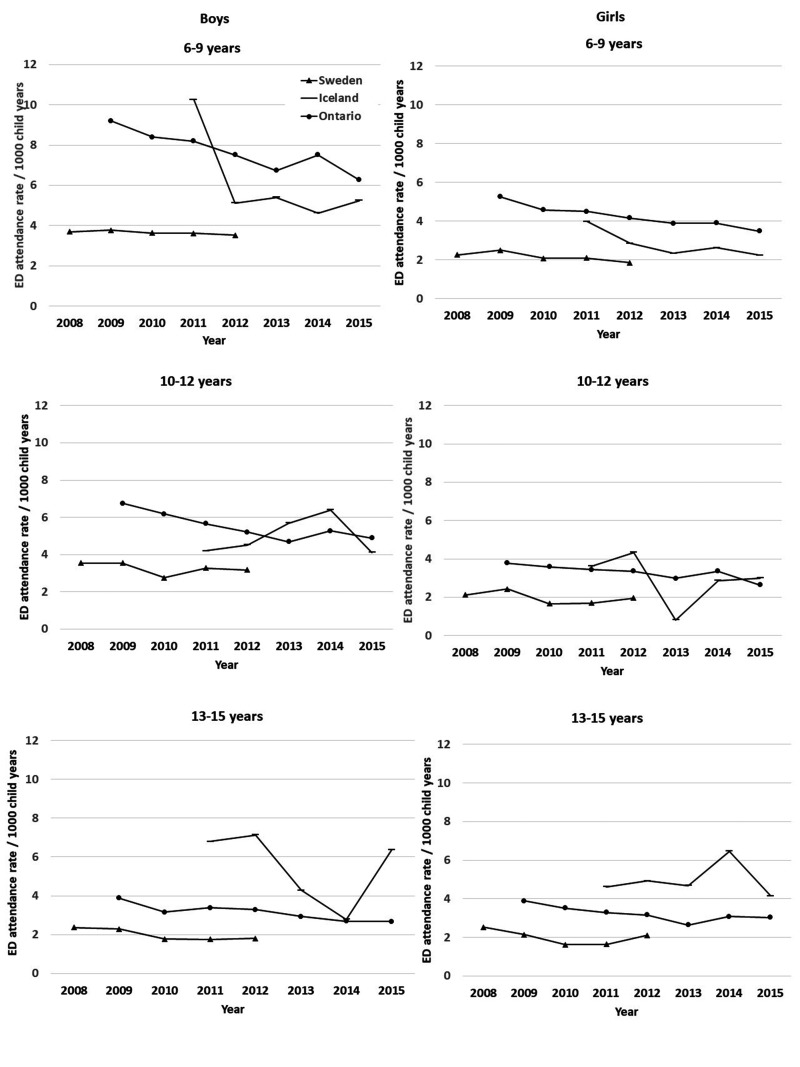
Emergency department attendance rates (per 1000 child years) by age, sex, jurisdiction and year.

Sweden consistently had the lowest rate of ED attendances over time, and across sex and age groups compared with Ontario and Iceland (Figure 3). Iceland had asthma admission rates that were only 32% of Sweden’s. However, if ED attendance rates for asthma were considered, Iceland had 75% higher ED attendance rates for asthma than Sweden (Table S3).

Zero-day admissions accounted for 56% of all admissions with a primary diagnosis of asthma in England. When we excluded 0-day admissions in England, asthma admission rates relative to Sweden’s decreased drastically (IRR = 4.61; 95% CI 4.30–4.94 vs 7.19; 95% CI 6.79–7.61 when including 0-day admissions in England - Figure S1 and Table S4).

**Figure 4.**
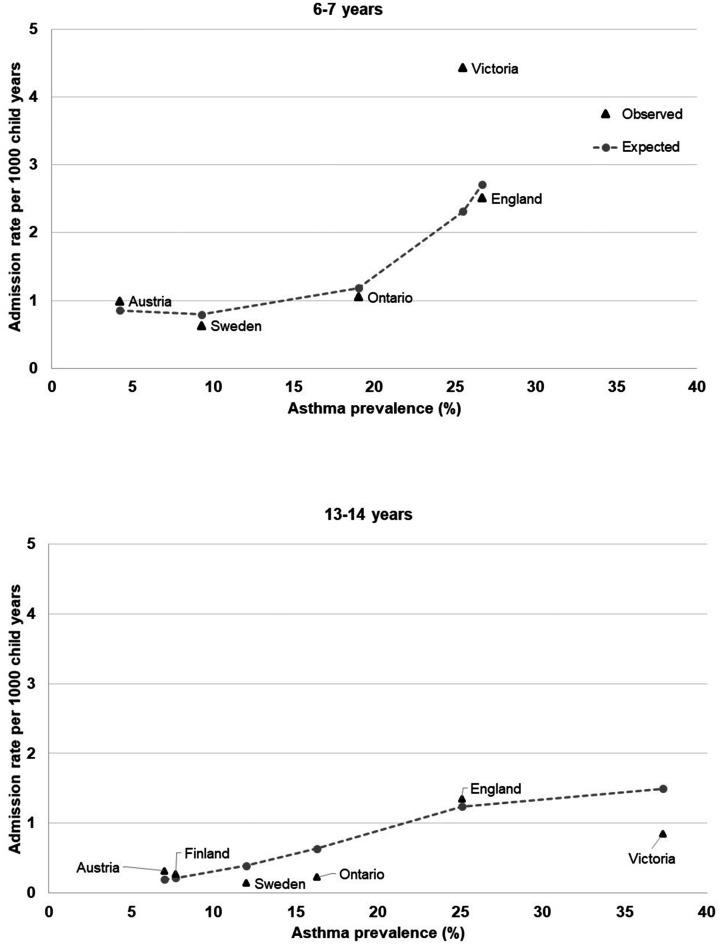
Observed and expected (from Poisson regression models) association between asthma prevalence from ISAAC^6^ and admission rates (with asthma recorded as primary diagnosis).

A quadratic term for asthma prevalence improved the fit of the Poisson regression models with asthma admissions modelled as a function of asthma prevalence (LR-test compared with linear term only for both age groups *p* < 0.001). We identified a positive relationship between ISAAC asthma prevalence rates and asthma primary diagnosis admission rates for 6–7 year-olds: when asthma prevalence increased above 10% there was a proportional increase in admission rates (Figure 4). For 13–14 year-olds, the relationship between admission rates and asthma prevalence was weaker, particularly for countries with a prevalence of <20%.

## Discussion

We found an eight-fold difference in asthma admission rates between jurisdictions. Iceland and Sweden had the lowest asthma admission rates, and England and Victoria the highest. These results were dependent on whether admissions had asthma recorded as the primary diagnosis or as any of the diagnoses recorded. Rates of ED attendance were nine to 50 times higher than for asthma admissions, and the relative ranking would have altered had we used ED attendances rather than admissions to compare jurisdictions. Asthma admission rates were positively associated with prevalence, particularly among children aged 6–7 years old.

The inter-country differences in asthma admission rates reported in our study are similar to those previously reported by the OECD for adults, with England and Australia having the highest rates, and Scandinavian countries the lowest.^2^

Unlike previous studies, however, our results highlight the importance of understanding the context of hospital administrative data collection across different settings. We identified substantial differences in rates of hospital admissions due to asthma depending on the definition used. Over half of admissions in England where asthma was recorded as a primary diagnosis were 0-day admissions. Further, admission rates in England were strongly dependent on whether asthma was recorded as a primary or any diagnosis. Differences were smaller in other jurisdictions. In 2004, England introduced Payment by Results, an activity-based hospital funding model of paying National Health Service (NHS) hospitals a tariff for each patient treated, based on the complexity of the patients’ condition or operation carried out.^25^ Hospitals therefore have an incentive to record comorbidities if present. However, similar systems of hospital reimbursement are also operating in a number of other jurisdictions in this study, including Sweden and Victoria (see Table S1). It therefore appears that the coding of asthma as a comorbidity leads to a higher reimbursement under the English Payment by Results system than in other activity-based systems. We recommend using admissions with asthma as a primary diagnosis only to compare the burden of asthma seen in hospitals, since coding of asthma as a comorbidity (any diagnosis) appears more sensitive to the model of hospital reimbursement used. Coding practices may also vary between jurisdiction due to other factors, including who enters the code (clinicians, or clinical coders based on patient notes), condition awareness or local coding guidelines.

We showed that asthma-related ED attendances were nine times higher in Sweden and Ontario, and fifty times higher in Iceland compared with asthma hospital admissions. Had we used ED attendance rates as an indicator of primary care quality, the relative performance of Sweden and Iceland would have been inverted compared with when asthma admissions were used. A previous study demonstrated that ED attendance rates in infants were comparable in England and Ontario whereas the probability of hospital admission was 2.5 times higher in England.^15^ The widely different admission rates among countries with relatively similar ED attendance rates can be explained by a number of factors affecting the decision to admit a child presenting to ED, including waiting time targets or availability of hospital beds. Individual-level data on all secondary care contacts (not just admissions) are required to more accurately profile asthma-related secondary care use.

We identified a positive relationship between asthma prevalence from ISAAC and asthma hospital admission rates particularly among 6–7 year-olds in higher prevalence jurisdictions. The ISAAC study team have previously reported positive associations between wheeze prevalence rates and asthma hospital admissions in children from both the first and third waves of ISAAC, particularly for 13–14 year-olds.^26^ These results highlight the basic challenge of interpreting hospital admission rates for asthma in isolation as an indicator of quality of care provided to children with asthma. Namely, asthma hospital admission rates are associated with asthma prevalence, particularly in higher prevalence countries (where asthma prevalence is greater than 10%) and among younger children. Therefore, high asthma admission rates in one country compared with another may simply reflect higher asthma prevalence, and not necessarily poor quality primary care for children with asthma.^27^ A number of factors have been associated with the prevalence and severity of asthma including socio-economic status, tobacco smoke exposure, and indoor and outdoor environments. In addition, the cost of medications to families have been shown to impact asthma exacerbation rates in the United States^28^ and Canada.^29^

We used routinely collected administrative hospital data from seven jurisdictions for this study. Each hospital database has complete area coverage, thus minimising selection biases. Since ICD-10 coding is used in hospital databases globally, international comparisons based on these datasets are more straightforward to perform. We were able to analyse admissions with asthma recorded as a primary diagnosis and admissions with asthma recorded as any diagnosis for five jurisdictions, and asthma ED attendances for three jurisdictions. This is, therefore, the most comprehensive study of secondary care contacts for asthma across multiple high-income jurisdictions to date.

## Limitations

There are several limitations to this study. First, we calculated asthma hospital admission and ED attendance rates using a population denominator of all children of a particular sex and age group. Ideally, we would have examined asthma admissions among the population of children diagnosed with asthma in each jurisdiction. In order to examine whether rates of hospital contacts for children with asthma vary between countries, data from registries, primary care or dispensing records could be used to derive estimates of the population of children by age and sex who have been diagnosed with, or are treated for, asthma. These data are currently only available for Ontario, which has primary care visit data, and Sweden, Iceland and Finland, which have community dispensing data. Common definitions to characterize the population of children with asthma, according to severity, in different jurisdictions would therefore need to be developed.

Second, in our comparison between ISAAC prevalence data and asthma admissions, we assumed asthma prevalence rates in localities participating in ISAAC Wave 3 surveys were representative of the whole country.^6^ In addition, although we used data from the last wave of ISAAC, these were collected between 2001 and 2003, and may not reflect current asthma prevalence across the different jurisdictions. For example, asthma prevalence in England has decreased since the early 2000s.^30^ Despite these challenges, ISAAC Wave 3 data was the most recent available data on asthma prevalence, collected using similar methodology and definitions, across the jurisdictions studied. Our study highlights the need for timely, internationally comparable estimates of asthma prevalence among children.

Third, not all indicators of asthma hospital contacts were available for all jurisdictions. We could not exclude 0-day admissions from our datasets from Ontario or Victoria. Data on asthma recorded as any diagnosis were not available from Austria or Iceland, and asthma ED attendances were only available from Iceland, Sweden and Ontario. The lack of data on ED attendances highlight that for this area of secondary care, data are not routinely collected, or if collected, diagnostic information or attendance reason is not recorded or not recorded using ICD-10 standardized coding. Further, due to small number of events, the observed rates were unstable in some jurisdictions, particularly Iceland.

High quality, affordable primary care remains a key component of asthma management in children to prevent exacerbations requiring ED attendances or hospital admissions. International comparisons of primary care quality for children with asthma could provide important information on how to improve care. However, in order to assess the quality of primary care for children with asthma using hospital admission rates, national coverage or nationally representative primary care or dispensing data linked to hospital admission data are required to identify resident children with asthma and estimate hospital admission rates among them using similar definitions of an asthma admission. Such an approach would also allow inter-country differences in asthma severity to be taken into account. Until such data are available, standardized and validated across a number of locations, international comparisons of asthma admission rates as an indicator of quality of primary care services should be interpreted with extreme caution.

## Conclusions

We identified large variations between countries in asthma hospital admission rates among children; the highest rates were found in Victoria and England. However, asthma admission rates were found to be highly sensitive to the definition used and associated with the underlying prevalence of asthma. Asthma hospital admissions should therefore not be used as a national indicator of quality of primary care for children with chronic conditions without careful consideration of definitions used and other, not necessarily health care related, drivers of observed differences.

## Supplemental Material

sj-pdf-1-hsr-10.1177_13558196211012732 - Supplemental material for Challenges of using asthma admission rates as a measure of primary care quality in children: An international comparison Click here for additional data file. Supplemental material, sj-pdf-1-hsr-10.1177_13558196211012732 for Challenges of using asthma admission rates as a measure of primary care quality in children: An international comparison by Irina Lut, Kate Lewis, Linda Wijlaars, Ruth Gilbert, Tiffany Fitzpatrick, Hong Lu, Astrid Guttmann, Sharon Goldfield, Shaoke Lei, Geir Gunnlaugsson, Stefán Hrafn Jónsson, Reli Mechtler, Mika Gissler, Anders Hjern and Pia Hardelid in Journal of Health Services Research & Policy
