# Supplementary material for: Challenges of using asthma admission rates as a measure of primary care quality in children: An international comparison
Source: J Health Serv Res Policy. 2021 Jul 28;26(4):251–62. doi: 10.1177/13558196211012732 (PMC8564239; doi:10.1177/13558196211012732)

Challenges of using asthma admission rates as a measure of primary care quality in children: an international comparison

Supplement 1: additional tables and figures

Table S1. Description of data sources, availability and definitions by jurisdiction

Jurisdiction	Hospital database	Denominator population – data source	Definition of hospital admission	Definition of primary diagnosis	Hospital reimbursement mechanism[1]	Outcomes available	Years of data available
**Sweden**	https://www.ncbi.nlm.nih.gov/pubmed/21658213<br><br>http://www.socialstyrelsen.se/register/halsodataregister/patientregistret/inenglish	http://www.socialstyrelsen.se/publikationer2003/2003-112-3<br>https://link.springer.com/article/10.1007%2Fs10654-016-0117-y	Overnight stay	• Not formally defined but understood to reflect the main cause of care at the time of discharge	• Hospitals are reimbursed through a mixture of global budgets from county councils, hospital activity based case payment systems, and patient contributions.	• Primary<br>• Any<br>• ED	2008-2012
**England**	https://digital.nhs.uk/data-services/hospital-episode-statistics	https://www.ons.gov.uk/peoplepopulationandcommunity/populationandmigration/populationestimates/datasets/populationestimatesforukenglandandwalesscotlandandnorthernireland	All finished consultant episodes with admission date <=1 day after previous discharge date were included in the same admission. All hospital activities allocated an NHS bed are counted as an admission, including day cases and admissions	• The main condition treated or investigated during the relevant episode of care or main symptom when no diagnosis	• All NHS hospitals are reimbursed through an activity based case payment system	• Primary<br>• Any	2009-2013

			with lengths of stay<1 day.				
**Finland**	https://thl.fi/en/web/thlfi-en/statistics/information-on-statistics/register-descriptions/care-register-for-health-care	http://pxnet2.stat.fi/PXWeb/pxweb/en/StatFin/StatFin__vrm__vaerak/?tablelist=true	Overnight stay	• Reflects the diagnosis or procedure accounting for the highest cost to the hospital (determined at discharge)	• Services are covered from user charges (<10%) and municipalities which make annual contracts with district hospitals based on an activity-based case payment system	• Primary<br>• Any	2009-2013
**Austria**	https://www.gesundheitsplanung.at/	http://statistik.at/web_de/services/statcube/index.html	Discharge after >1day (night included) admission	• Reflects the diagnosis or procedure accounting for the highest cost to the hospital (determined at discharge)	• State level funding (there are 9 states in Austria) financed by a mixture of funds, including the state government through activity based allocation system insurance companies and others.	• Primary	2010-2014

**Iceland**	https://www.landlaeknir.is/tolfraedi-og-rannsoknir/gagnasofn/gagnasafn/item12464/Vistunarskra-heilbrigdisstofnana. See also information in English: https://www.landlaeknir.is/english/statistics/health-care-services/hospitals/	https://statice.is/statistics/population/inhabitants/	At least one night	• Reflects the main reason for care (laboratory analysis or treatment) but not required. If an external injury is the cause for admission, two codes are to be provided.	• No direct payment for specific services ; hospitals receive an annual budget by the government and are state-run	• Primary<br>• ED	2011-2015
**Ontario**	https://www.cihi.ca/en	https://www.ontario.ca/data/registered-persons-database-rpdb	Finished consultant episode with admission date >=1 day after previous discharge	• Reflects the main reason for admission; what contributes to the greatest length of stay or use of resources	• Hospitals are funded through a mixed model including on global funding; health based allocation model (predicted costs based on patient demographics); and quality-based procedure model (payment bundles	• Primary<br>• Any<br>• ED	2009-2015

					based on types and quantities of patients treated).		
**Victoria**	https://www2.health.vic.gov.au/hospitals-and-health-services/data-reporting/health-data-standards-systems/data-collections/vaed	http://www.abs.gov.au/AUSSTATS/abs@.nsf/Lookup/3101.0Main+Features1Sep%202018?OpenDocument	Follows a clinical decision that a patient requires a hospital stay (could be same-day, overnight or multi-day care or treatment) https://www2.health.vic.gov.au/about/publications/policiesandguidelines/Victorian-Admitted-Episodes-Dataset-manual-2019-2020 VAED Manual, Section 2	• Reflects the main reason for an episode of admitted patient care, residential care or attendance at a healthcare establishment	• Public hospitals are reimbursed through an activity based case payment system	• Primary<br>• Any	2009-2012

1. Mihailovic N, Kocic S, Jakovljevic M. Review of Diagnosis-Related Group-Based Financing of Hospital Care. *Health services research and managerial epidemiology* 2016;3:. doi: 10.1177/2333392816647892

Table S2: Incidence rate ratios (IRRs)* with 95% confidence intervals (CIs) for asthma admission rates by jurisdiction compared to Sweden (baseline), according to whether asthma was recorded as the primary or any diagnosis

	**IRR (95% CI)**	
**Jurisdiction**	**Primary diagnosis**	**Any diagnosis**
Sweden	1 (baseline)	1 (baseline)
England	7.19 (6.79, 7.61)	10.84 (10.45, 11.23)
Finland	1.44 (1.33, 1.56)	1.22 (1.16, 1.29)
Austria	2.55 (2.38, 2.73)	No data
Iceland	0.32 (0.20, 0.51)	No data
Ontario	1.94 (1.83, 2.07)	1.24 (1.19, 1.29)
Victoria	8.20 (7.71, 8.72)	3.58 (3.43, 3.74)

*Adjusted for age group and sex

Table S3. Asthma admission/ED attendance rate by indicator, jurisdiction, age & sex with 95% confidence intervals (CIs)

Jurisdiction	Gender	Age (years)	Asthma admission rate / 1000 child years (95% CI) Primary diagnosis	Asthma admission rate / 1000 child years (95% CI) any diagnosis	ED attendance rate / 1000 child years (95% CI)
**Austria**	Boys	6 to 9	1.09 (1.03-1.17)	-	-
		10 to 12	0.91 (0.84-0.99)	-	-
		13 to 15	0.46 (0.41-0.51)	-	-
	Girls	6 to 9	0.61 (0.56-0.67)	-	-
		10 to 12	0.37 (0.33-0.43)	-	-
		13 to 15	0.48 (0.42-0.53)	-	-
**England**	Boys	6 to 9	2.96 (2.91-3.00)	8.92 (8.85-8.99)	-
		10 to 12	2.07 (2.03-2.11)	8.19 (8.10-8.27)	-
		13 to 15	1.27 (1.24-1.31)	7.80 (7.72-7.88)	-
	Girls	6 to 9	1.84 (1.81-1.87)	5.44 (5.38-5.50)	-
		10 to 12	1.60 (1.56-1.64)	5.54 (5.47-5.61)	-
		13 to 15	1.38 (1.35-1.42)	7.69 (7.61-7.77)	-
**Finland**	Boys	6 to 9	0.72 (0.66-0.79)	1.40 (1.31-1.50)	-
		10 to 12	0.37 (0.31-0.43)	0.843 (0.76-0.93)	-
		13 to 15	0.27 (0.23-0.32)	0.68 (0.61-0.76)	-
	Girls	6 to 9	0.38 (0.33-0.43)	0.74 (0.67-0.81)	-
		10 to 12	0.23 (0.19-0.28)	0.54 (0.48-0.62)	-
		13 to 15	0.21 (0.17-0.26)	0.55 (0.48-0.62)	-
**Iceland**	Boys	6 to 9	0.24 (0.12-0.44)	-	5.70 (4.50-6.48)
		10 to 12	0.12 (0.03-0.32)	-	4.98 (5.24-5.81)
		13 to 15	0.06 (0-0.22)	-	5.47 (4.70-6.34)

	Girls	6 to 9	0.02 (0-0.13)	-	2.79 (2.32-3.34)
		10 to 12	0 (0-0.12)	-	2.94 (2.37-3.60)
		13 to 15	0.03 (0-0.19)	-	4.87 (4.12-5.74)
**Ontario**	Boys	6 to 9	0.99 (0.95-1.03)	1.46 (1.41-1.52)	7.68 (7.56-7.79)
		10 to 12	0.51 (0.48-0.55)	0.82 (0.78-0.87)	5.53 (5.42-5.64)
		13 to 15	0.21 (0.19-0.23)	0.49 (0.46-0.52)	3.15 (3.07-3.23)
	Girls	6 to 9	0.66 (0.63-0.69)	0.96 (0.92-1.00)	4.24 (4.15-4.33)
		10 to 12	0.35 (0.32-0.38)	0.56 (0.52-0.60)	3.31 (3.22-3.40)
		13 to 15	0.19 (0.17-0.21)	0.50 (0.47-0.54)	3.24 (3.16-3.33)
**Sweden**	Boys	6 to 9	0.48 (0.44-0.52)	0.94 (0.87-1.00)	3.65 (3.53-3.77)
		10 to 12	0.24 (0.20-0.28)	0.69 (0.62-0.75)	3.26 (3.12-3.40)
		13 to 15	0.14 (0.11-0.17)	0.59 (0.54-0.65)	2.01 (1.91-2.12)
	Girls	6 to 9	0.32 (0.28-0.36)	0.62 (0.57-0.68)	2.15 (2.06-2.25)
		10 to 12	0.15 (0.12-0.19)	0.49 (0.44-0.55)	1.97 (1.86-2.08)
		13 to 15	0.17 (0.14-0.21)	0.61 (0.55-0.67)	2.03 (1.92-2.14)
**Victoria**	Boys	6 to 9	4.1 (3.98-4.32)	4.47 (4.29-4.65)	-
		10 to 12	1.98 (1.85-2.12)	2.21 (2.07-2.36)	-
		13 to 15	0.92 (0.83-1.02)	1.06 (0.97-1.17)	-
	Girls	6 to 9	2.72 (2.58-2.86)	3.03 (2.88-3.18)	-
		10 to 12	1.46 (1.34-1.59)	1.61 (1.48-1.74)	-
		13 to 15	0.99 (0.90-1.10)	1.11 (1.01-1.23)	-

Table S4: Incidence rate ratios (IRRs)* with 95% confidence intervals (CIs), asthma admission rates by jurisdiction to Sweden, according to with asthma recorded as the primary diagnosis and no zero day admissions

	IRR (95% CI)
**Jurisdiction**	**Primary diagnosis**
Sweden	1 (baseline)
England	4.61 (4.30-4.94)
Finland	1.43 (1.32, 1.55)
Austria	2.53 (2.36, 2.70)
Iceland	0.32 (0.20, 0.51)

*Adjusted for age group and sex

Figure S1. Asthma admission rates (by age and gender) including and excluding zero day admissions in England

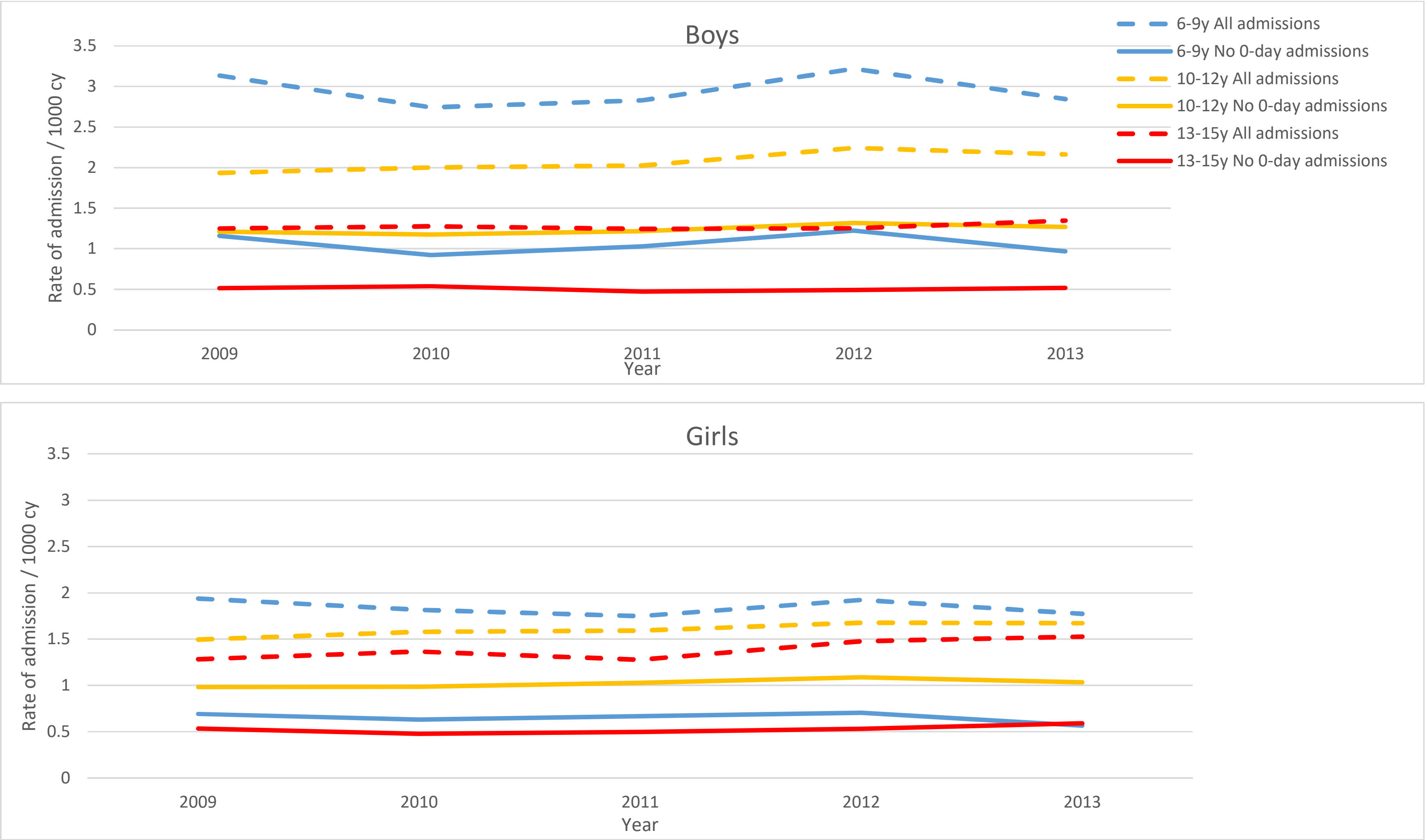

Supplement: sj-pdf-1-hsr-10.1177_13558196211012732 - Supplemental material for Challenges of using asthma admission rates as a measure of primary care quality in children: An international comparison [file sj-pdf-1-hsr-10.1177_13558196211012732.pdf]
